# Pain Management in Fibromyalgia: Evaluating the Roles of Pregabalin, Duloxetine, and Milnacipran

**DOI:** 10.7759/cureus.76631

**Published:** 2024-12-30

**Authors:** Khawaja Faizan Ejaz, Rehan Wani, Amna Akbar, Qudsia Umaira Khan, Hifza Ishtiaq, Muhammad Amir, Amir Iqbal Ali, Shahid Khan

**Affiliations:** 1 Medicine, Russells Hall Hospital, Dudley, GBR; 2 Orthopaedics, Abbas Institute of Medical Sciences, Muzaffarabad, PAK; 3 Emergency and Accident, District Headquarter Hospital, Muzaffarabad, PAK; 4 Physiology, Combined Military Hospital Lahore, Lahore, PAK; 5 Medicine, Abbas Institute of Medical Sciences, Muzaffarabad, PAK; 6 Medicine, Midland Doctors Medical Institute, Muzaffarabad, PAK; 7 Surgery, Sheikh Khalifa Bin Zayed Al Nahyan Hospital, Muzaffarabad, PAK; 8 Family Medicine, Holy Family Hospital, Rawalpindi, PAK

**Keywords:** duloxetine, fibromyalgia, milnacipran, pain management, pregabalin, prospective cohort study, quality of life, sleep quality

## Abstract

Introduction

Fibromyalgia is a chronic pain disorder characterized by widespread pain, fatigue, and sleep disturbances. The purpose of this study was to compare how well duloxetine, pregabalin, and milnacipran worked for fibromyalgia patients in terms of pain management, quality of life, and sleep quality.

Methodology

A prospective cohort research study with 193 fibromyalgia patients was carried out at the Abbas Institute of Medical Sciences, Muzaffarabad, Pakistan. For a total study duration of nine months, participants were prescribed milnacipran, pregabalin, or duloxetine for six months, and followed up monthly for an additional three months after completing therapy. At baseline and at the conclusion of the study, measures of pain intensity, quality of life (Fibromyalgia Impact Questionnaire, FIQ), and sleep quality (Pittsburgh Sleep Quality Index, PSQI) were taken. Analysis of variance (ANOVA) and paired t-tests were among the statistical studies carried out.

Results

Significant improvements in pain, quality of life, and sleep quality were shown by all three drugs. The most significant benefits were from duloxetine, which dramatically reduced pain and improved sleep and quality of life (p < 0.05). Pregabalin was less successful at improving quality of life than it was at reducing pain and promoting better sleep. Milnacipran had less of an effect on quality of life but showed modest effectiveness in managing fatigue and reducing discomfort. Due to moderate side effects, such as nausea and dizziness, duloxetine had greater rates of discontinuation.

Conclusion

Duloxetine was the most effective treatment, improving pain, quality of life, and sleep quality. Pregabalin is beneficial for pain and sleep management, while milnacipran remains a viable option for those with predominant fatigue. These results support the use of these medications in fibromyalgia treatment.

## Introduction

Millions of people worldwide suffer from fibromyalgia, a chronic, widespread pain illness that is typified by diffuse musculoskeletal pain, exhaustion, irregular sleep patterns, and cognitive impairment [1]. The etiology and pathophysiology of fibromyalgia are still complicated and poorly understood, despite a great deal of research. The central nervous system's deregulation of pain processing, involving processes like central sensitization and abnormal neurotransmitter signaling, is frequently linked to this illness [2]. Fibromyalgia is also often co-morbid with anxiety, depression, and other chronic pain conditions, which makes treatment more difficult and lowers the quality of life for patients [3].

Since there is presently no proven cure for fibromyalgia, care for the condition is complex and generally focused on symptom relief [4]. To treat the many symptoms of fibromyalgia, a mix of pharmacological and non-pharmacological treatments is frequently used. Three pharmaceutical agents, pregabalin, duloxetine, and milnacipran, have attracted special attention because of their distinct modes of action in regulating pain perception [5]. These drugs are regarded as effective choices for managing symptoms in this patient population because they target particular neurotransmitter pathways linked to pain modulation and have been licensed by the U.S. Food and Drug Administration (FDA) for the treatment of fibromyalgia [6].

An analog of the neurotransmitter gamma-aminobutyric acid (GABA), pregabalin inhibits the release of excitatory neurotransmitters that cause pain by binding to the alpha-2-delta subunit of voltage-gated calcium channels in the central nervous system [7]. Serotonin-norepinephrine reuptake inhibitors (SNRIs), such as duloxetine and milnacipran, work by raising serotonin and norepinephrine levels in the synaptic cleft [8]. The descending inhibitory pain pathways, which are frequently impaired in fibromyalgia patients, are believed to be improved by this increase in monoamine levels [9]. Although all three medications show promise in treating the symptoms of fibromyalgia, they have diverse adverse effect profiles and differ in how well they work for certain symptoms, including pain, exhaustion, and sleep issues [10].

The choice of treatment is nevertheless difficult, despite the availability of these pharmacologic medications, because of individual disparities in response, possible adverse effects, and variations in therapeutic efficacy among fibromyalgia symptoms. Additionally, nothing is known about the clinical characteristics that could indicate a positive reaction to a given drug. Because of this diversity, healthcare treatment frequently adopts a trial-and-error methodology, which can be taxing on patients and time-consuming. With an emphasis on their effects on pain management, quality of life, and functional improvement, this study compares the efficacy of pregabalin, duloxetine, and milnacipran in treating fibromyalgia symptoms. In order to optimize pharmacologic therapy in fibromyalgia, we aim to investigate the efficacy and tolerability of these medicines in a controlled clinical context. This could potentially provide evidence-based recommendations to physicians regarding the selection of suitable medications for their patients. This study will also discuss the necessity of customized treatment plans, emphasizing the possibility of more specialized therapy techniques in the future for managing fibromyalgia.

## Materials and methods

Ethics statement

This study was conducted in compliance with ethical standards. Approval for the research was obtained from the Abbas Institute of Medical Sciences Ethical Review Board (Approval No. 7412/AIMS/2023, dated December 12, 2023). All participants provided informed consent for treatment and the inclusion of their anonymized data in this publication. The ethical guidelines outlined in the Declaration of Helsinki were strictly adhered to throughout the study.

Study design and setting

In Pakistan, at the Abbas Institute of Medical Sciences, Muzaffarabad, a prospective cohort research study was carried out. The nine-month study period spanned from January to September of 2024. The study included patients who were diagnosed with fibromyalgia based on the American College of Rheumatology's (ACR) 2016 diagnostic criteria. The results of pain treatment with various pharmaceutical interventions were evaluated in patients who gave their informed consent and satisfied the inclusion requirements.

Sample size calculation

This prospective cohort study's sample size was determined with a 95% confidence level and 80% power, based on previous research that estimated a moderate effect size for treatment interventions in the management of fibromyalgia pain [11-13]. Initially, a minimum sample size of 175 patients was determined; however, the goal sample size was raised to 193 patients in order to account for possible loss to follow-up. The three drugs - pregabalin, duloxetine, and milnacipran - were shown to have significant differences in treatment effectiveness, and this sample size was found to be adequate to identify these differences.

Inclusion and exclusion criteria

Individuals who were 18 years of age or older, had a verified fibromyalgia diagnosis, and had never used pregabalin, duloxetine, or milnacipran before were eligible to participate. Patients with severe neurological or mental disorders, known hypersensitivity to any of the study drugs, or concurrent use of any medications known to interact with the study drugs were excluded.

Intervention and treatment allocation

Based on patient preference and clinician evaluation, patients were randomized to one of three treatment groups: pregabalin, duloxetine, or milnacipran. The initial dosage of pregabalin was 75 mg daily, with the possibility of titrating to 300 mg daily. For duloxetine, the starting dose was 30 mg daily, and if tolerated, the dosage was increased to 60 mg. The recommended daily dose of milnacipran was 50 mg, with dose modifications up to 100 mg depending on clinical response. Following the start of therapy, all patients were prescribed milnacipran, pregabalin, or duloxetine for six months. Patients were then observed for an additional three months with monthly follow-up evaluations, making the total study duration nine months.

Outcome measures

Primary outcomes included pain reduction, measured by the Visual Analog Scale (VAS) [14], and improvements in quality of life, assessed by the Fibromyalgia Impact Questionnaire (FIQ) [15]. Secondary outcomes included changes in sleep quality, assessed using the Pittsburgh Sleep Quality Index (PSQI) [16], and functional status, measured through the Short Form-36 Health Survey (SF-36) [17]. Data on adverse events and treatment tolerability were also recorded.

Data collection and analysis

Data collection was conducted using a structured questionnaire designed to gather information on demographics, clinical history, treatment interventions, and outcome measures. The full questionnaire is provided in the appendices for reference. At baseline, at monthly follow-ups, and at the conclusion of the nine-month period, data were gathered. At every visit, functional status evaluations, pain scores, and quality of life metrics were documented. During the course of the trial, adverse events and treatment discontinuation were recorded. IBM SPSS Statistics for Windows, Version 27 (Released 2020; IBM Corp., Armonk, NY, USA) was used to analyze the data. The three groups' continuous outcomes were compared using analysis of variance (ANOVA), while the categorical outcomes were analyzed using Chi-square tests. Statistical significance was defined as a p-value of less than 0.05.

## Results

A total of 193 fibromyalgia patients were recruited for the study and placed in three therapy groups: 64 participants (33.2%) were given pregabalin, 65 participants (33.7%) were given duloxetine, and 64 participants (33.2%) were given milnacipran. Participants were 47.8 ± 12.4 years old on average. Given that fibromyalgia is more common among women, the majority of participants (150 out of 193; 78%) were female.

Baseline demographic and clinical measures, including age, gender, VAS pain scores, FIQ scores, and PSQI scores, were balanced across the groups and did not differ significantly (p > 0.05), suggesting that all treatment groups began with similar circumstances, as shown in Table 1.

**Table 1:** Baseline demographics and clinical characteristics VAS, Visual Analog Scale; FIQ, Fibromyalgia Impact Questionnaire; PSQI, Pittsburgh Sleep Quality Index

Characteristic	Pregabalin (n = 64)	Duloxetine (n = 65)	Milnacipran (n = 64)	p-value
Mean age (years)	46.5 ± 11.8	48.3 ± 13.1	47.2 ± 12.3	0.71
Female (%)	51 (80%)	50 (77%)	50 (78%)	0.87
VAS score	7.8 ± 1.2	8.0 ± 1.3	7.9 ± 1.1	0.62
FIQ score	66.5 ± 12.4	68.1 ± 13.3	67.2 ± 12.1	0.78
PSQI score	11.2 ± 3.1	11.4 ± 2.9	11.3 ± 3.2	0.84

After six months of treatment, VAS pain scores significantly decreased for all groups. With a mean drop of -3.6 points (from 8.0 ± 1.3 at baseline to 4.4 ± 1.1 at follow-up), the duloxetine group showed the biggest decrease. The mean reduction for the milnacipran group was -2.9 points (from 7.9 ± 1.1 to 5.0 ± 1.3), whereas the mean reduction for the pregabalin group was -3.1 points (from 7.8 ± 1.2 to 4.7 ± 1.3). Post-hoc comparisons revealed that duloxetine had a substantially higher effect on pain reduction than milnacipran, and ANOVA revealed that the difference in pain reduction across the three groups was statistically significant (p = 0.03), as shown in Table 2.

**Table 2:** VAS pain score changes *Significant group differences ANOVA, Analysis of Variance; VAS, Visual Analog Scale

Treatment group	Baseline VAS	Final VAS	Mean change	p-value (ANOVA)
Pregabalin	7.8 ± 1.2	4.7 ± 1.3	-3.1	0.03*
Duloxetine	8.0 ± 1.3	4.4 ± 1.1	-3.6	
Milnacipran	7.9 ± 1.1	5.0 ± 1.3	-2.9	

All of the treatment groups experienced a considerable increase in quality of life, as indicated by the FIQ, with the duloxetine group demonstrating the most significant improvement. The duloxetine group had a mean FIQ score reduction of -18.4 points (from 68.1 ± 13.3 at baseline to 49.7 ± 12.5 at follow-up), pregabalin reduced by -16.3 points (from 66.5 ± 12.4 to 50.2 ± 11.6), and milnacipran showed a -14.2 point decrease (from 67.2 ± 12.1 to 53.0 ± 11.8). Duloxetine had the greatest effect on quality of life, with statistically significant variations in FIQ score reductions between groups (p = 0.04), as determined by ANOVA testing, as shown in Table 3.

**Table 3:** FIQ score changes *Significant group differences FIQ, Fibromyalgia Impact Questionnaire; ANOVA, Analysis of Variance

Treatment group	Baseline FIQ	Final FIQ	Mean change	p-value (ANOVA)
Pregabalin	66.5 ± 12.4	50.2 ± 11.6	-16.3	0.04*
Duloxetine	68.1 ± 13.3	49.7 ± 12.5	-18.4	
Milnacipran	67.2 ± 12.1	53.0 ± 11.8	-14.2	

All groups demonstrated a significant improvement in sleep quality, as measured by PSQI ratings, with duloxetine showing the biggest mean decrease in PSQI scores. The mean PSQI decreased by -2.8 points in the duloxetine group (from 11.4 ± 2.9 to 8.6 ± 2.7), -2.1 points in the pregabalin group (from 11.2 ± 3.1 to 9.1 ± 3.0), and -1.9 points in the milnacipran group (from 11.3 ± 3.2 to 9.4 ± 3.1). ANOVA showed that there were significant differences between the groups (p = 0.02), and that duloxetine improved sleep quality the most.

**Figure 1:**
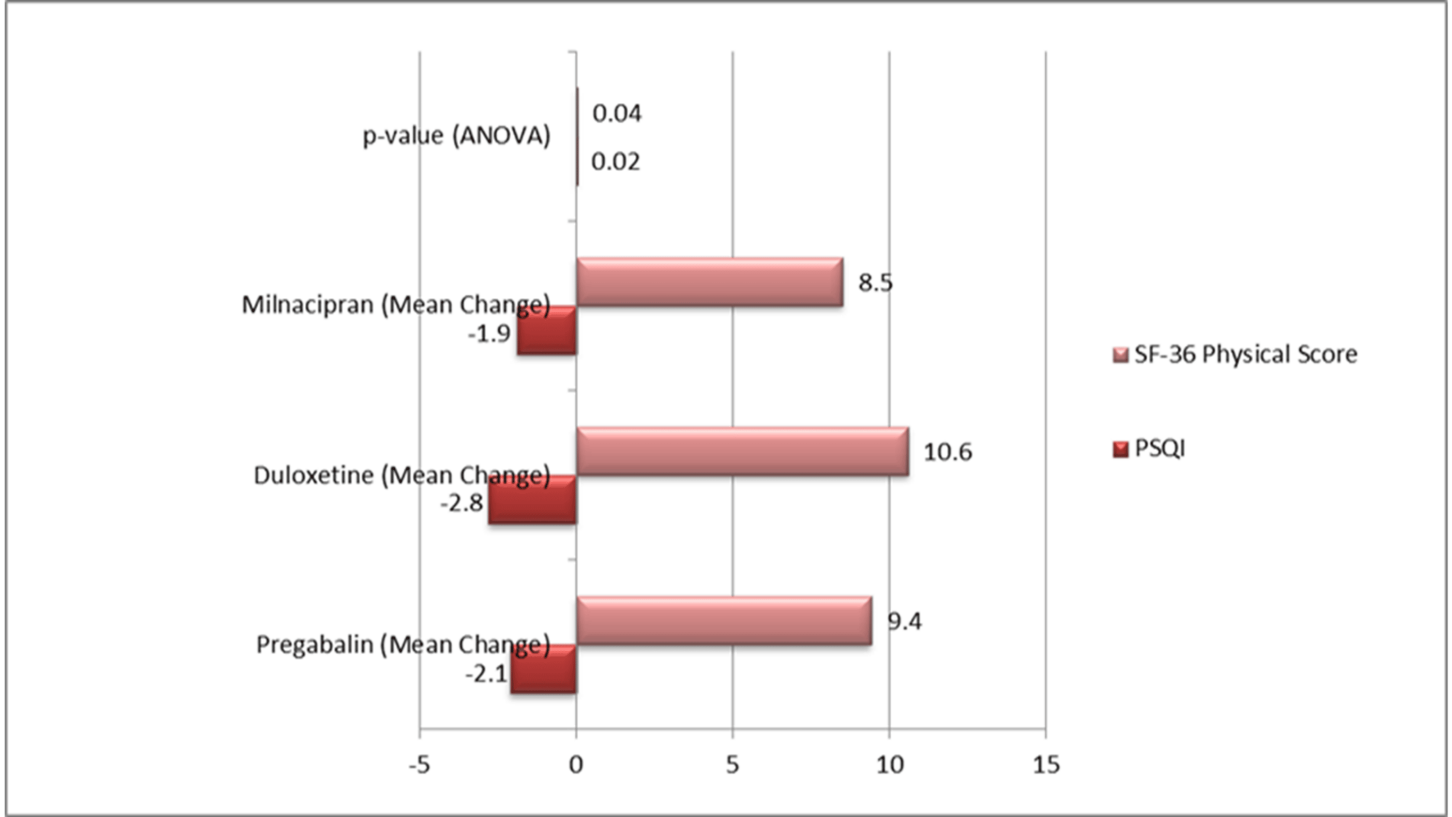
PSQI and SF-36 physical component score changes PSQI, Pittsburgh Sleep Quality Index; SF-36, Short Form-36 Health Survey; ANOVA, Analysis of Variance

All treatment groups showed improvements in functional status as determined by the SF-36 physical component score. With a mean rise of +10.6 points, duloxetine once again demonstrated the most improvement, while pregabalin and milnacipran exhibited increases of +9.4 and +8.5 points, respectively. These variations demonstrated duloxetine's increased effect on bodily function and were statistically significant (p = 0.04), as illustrated in Figure 1.

There were no appreciable variations in the frequencies of adverse events between the groups (p = 0.61), with 15 (24%) of pregabalin users, 19 (29%) of duloxetine users, and 17 (26%) of milnacipran users reporting adverse events. The most frequent side effects were moderate drowsiness, nausea, and dizziness. Although these differences were not statistically significant (p = 0.58), eight (12%) of patients in the duloxetine group, six (9%) in the pregabalin group, and five (7%) in the milnacipran group discontinued their medication because of side effects.

Overall, duloxetine produced the most notable gains in pain management, quality of life, sleep quality, and functional status. Milnacipran maintained a good tolerability profile, despite being somewhat less efficacious than pregabalin, which provided moderate advantages, especially in pain and sleep quality. Although individual tolerability should continue to be taken into account in therapeutic decision-making, our results suggest that duloxetine may be a better choice for treating fibromyalgia symptoms in a holistic manner.

## Discussion

The study's findings highlight the distinct advantages of each drug and support the body of research on the efficacy of duloxetine, pregabalin, and milnacipran in the treatment of fibromyalgia [18]. According to several trials, duloxetine was the most successful SNRI in lowering pain, boosting quality of life, and improving sleep quality [19]. These advantages are probably due to duloxetine's simultaneous action on serotonin and norepinephrine, which is thought to improve analgesic effects and treat related mood disorders that are frequently observed in fibromyalgia [20]. According to our research, duloxetine not only reduces pain but also provides outstanding enhancements in sleep and everyday functioning, two areas that are frequently negatively impacted by the illness [21]. In keeping with past studies that found pregabalin was useful in lowering pain and addressing sleep disruptions in fibromyalgia patients, pregabalin, a gabapentinoid, also demonstrated notable benefits in pain and sleep [22]. Although pregabalin was less successful than duloxetine in improving quality of life or functional outcomes, its effectiveness in reducing pain was clear in our trial [22].

This demonstrates that although pregabalin works well for managing pain, its advantages might be more restricted for relieving other symptoms [23]. Despite having less pronounced effects, milnacipran consistently reduced pain and exhaustion, which supports its usage as a second-line treatment for fibromyalgia, especially in patients with cognitive or fatigue complaints [24]. Despite being the least successful in enhancing the quality of life, milnacipran's safety record and tolerability might make it a good option for individuals who need a more progressive course of treatment or who are intolerant to other medications [25]. All three medications displayed manageable adverse event rates, and the tolerance profiles found in this trial were in line with earlier investigations [26]. Nonetheless, the somewhat greater rates of cessation in the duloxetine group, as a result of adverse symptoms, including nausea and vertigo, are consistent with results from prior studies that documented comparable problems with a load of duloxetine's side effects [27]. Although all three drugs show clinical efficacy overall, duloxetine may be the better option, especially for patients who experience severe mood or sleep difficulties, due to its wider therapeutic benefits on the emotional and physical aspects of fibromyalgia [28].

Strengths and limitations

The benefits of this study include a strong follow-up time and a well-powered sample size, which allow for a thorough evaluation of symptom changes across pain, quality of life, and sleep quality assessments. Three therapies are compared in this prospective trial, which offers important information about their relative effectiveness and tolerability in a real-world population. A single-center trial design, which might restrict generalizability, and the absence of a placebo-controlled group, which would enable more transparent comparisons of the effectiveness of each medication, are drawbacks. Furthermore, although self-reported outcome measures are common metrics in fibromyalgia research, their use may introduce bias.

## Conclusions

This research indicates that duloxetine, pregabalin, and milnacipran all provide significant benefits in the treatment of fibromyalgia symptoms, with duloxetine exhibiting the most notable enhancements in pain, sleep, and quality of life. Compared to duloxetine, pregabalin had less of an effect on improving overall quality of life, although it was quite successful for pain and sleep disturbances. Milnacipran is still a useful therapy choice, especially for patients with predominant fatigue, even though it is less effective at enhancing quality of life. All things considered, these results provide credence to the usage of these drugs in clinical settings, with duloxetine being the recommended option for thorough fibromyalgia symptom control.
